# A FRET Based Two-Photon Fluorescent Probe for Visualizing Mitochondrial Thiols of Living Cells and Tissues

**DOI:** 10.3390/s20061746

**Published:** 2020-03-21

**Authors:** Zhengkun Liu, Qianqian Wang, Hao Wang, Wenting Su, Shouliang Dong

**Affiliations:** 1 Institute of Biochemistry and Molecular Biology, School of Life Sciences, Lanzhou University, 222 Tianshui South Road, Lanzhou 730000, China; liuzhk14@lzu.edu.cn (Z.L.); wangqq16@lzu.edu.cn (Q.W.); wangh2017@lzu.edu.cn (H.W.); suwt18@lzu.edu.cn (W.S.); 2 Key Laboratory of Preclinical Study for New Drugs of Gansu Province, Lanzhou University, 222 Tianshui South Road, Lanzhou 730000, China

**Keywords:** mitochondrial thiols, fluorescent probe, FRET, two-photon

## Abstract

Glutathione (GSH) is the main component of the mitochondrial thiol pool and plays key roles in the biological processes. Many evidences have suggested that cysteine and homocysteine also exist in mitochondria and are interrelated with GSH in biological systems. The fluctuation of the levels of mitochondrial thiols has been linked to many diseases and cells’ dysfunction. Therefore, the monitoring of mitochondrial thiol status is of great significance for clinical studies. We report here a novel fluorescence resonance energy transfer based two-photon probe MT-1 for mitochondrial thiols detection. MT-1 was constructed by integrating the naphthalimide moiety (donor) and rhodamine B (accepter and targeting group) through a newly designed linker. MT-1 shows a fast response, high selectivity, and sensitivity to thiols, as well as a low limit of detection. The two-photon property of MT-1 allows the direct visualization of thiols in live cells and tissues by two-photon microscopy. MT-1 can serve as an effective tool to unravel the diverse biological functions of mitochondrial thiols in living systems.

## 1. Introduction

In mammalian cells, mitochondrial thiols, mainly Glutathione (GSH), is the most abundant non-protein thiol and plays a key role in the control of oxidative stress in redox homeostasis [1]. Mitochondria generates most of the cellular energy by means of oxidative phosphorylation, and also contributes to the main production of cellular reactive oxygen species (ROS) [2,3]. High ROS levels in mitochondria can cause oxidation of proteins, lipids, and other biomolecules, connecting to apoptosis and necrosis [4,5,6]. Mitochondrial GSH pool is a critical antioxidant reservoir within cells that involves the interconversion of reduced sulfhydryl (GSH) and oxidized disulfide (GSSG) forms. Besides, GSH is associated with many other mitochondrial functions, such as gene regulation, and signal transduction [7]. Therefore, the fluctuation in the levels of mitochondrial thiols is very important and has been linked to mitochondria’s functions and dysfunctions [8,9]. Although GSH is the major biological thiol in mitochondria, cysteine (Cys), homocysteine (Hcy) and likely other short peptide thiols are also present in mitochondria [10,11,12,13]. Many pieces of evidence have suggested that GSH and Cys/Hcy levels are interrelated in biological systems [14,15,16,17]. Therefore, an overall detection of mitochondrial thiols is of great importance in understanding the physiological mechanism of mitochondrial thiols and diagnosis of many diseases.

Currently, several methods including mass spectrometry [18], UV-vis assays [19], high-performance liquid chromatography [20], and electrochemical analysis [21] have been applied for thiols measurement. Among various methods, fluorescent probes have emerged as the most common imaging strategy due to its simplicity, high sensitivity and non-destructive advantages [22,23]. In the past ten years, a large number of fluorescent probes for cell thiols detection have been greatly developed and many have been well reviewed [24,25]. Fluorescent probes targeting mitochondria for mitochondrial thiols detection were also developed, and most of them were focused on the selective detection of GSH [26,27,28,29,30,31], Cys [11,12,32,33,34,35,36] or other thiols [37] based on preferred specific reaction sites. However, the fluorescent probes which can simultaneously react with GSH, Cys/Hcy, and other small molecule thiols for total mitochondrial thiols detection are still rare. As the best of our knowledge, there are only five probes reported for total mitochondrial thiols detection which were briefly reviewed hereinafter [38,39,40,41,42]. Fluorescent probes for mitochondrial thiols are mainly composed of three parts: (1) the fluorophores determining spectral properties; (2) the mitochondrial targeting group, mostly cationic; (3) the thiols reaction sites responsible for selectivity and sensitivity of probes. Two-photon microscopy (TPM) has become an important imaging tool for its low background signal, deeper tissue penetration, high signal-to-noise ratio, and better three-dimensional imaging compared to conventional confocal microscopy using one-photon excitation [43,44]. Indeed, as shown in Table S1, for these five reported probes, probe 1 [38], 2 [39], and 4 [40] adopted two-photon fluorophores. Although the excitation wavelengths were all around 740 nm, the emission wavelengths were slightly short (545 nm for probe 1, 442 nm for probe 2, and 482 nm for probe 4), thus limited their penetration abilities for the emitted light. The rest two probes (probe 3 [42] and 5 [41]) were one-photon and showed more redshift emission wavelengths, yet with much shorter excitation wavelengths. As to the mitochondria-targeting moieties, probe 1 and 2 equipped additional cationic triphenylphosphonium group [45] while probe 4 with addition imidazolium group. The remaining two probes took the advantages of the cationic properties of fluorophores itself and used them as mitochondria-targeting groups simultaneously (probe 3 with rhodamine cationic and probe 5 with bodipy cationic), therefore, the complexity of the probe molecules and the difficulty of synthesis were reduced. The reaction sites for thiols of five probes were also different. Classic disulfide linkage was adopted by probe 1 and showed a relatively slow response to biothiols. Arysulfonyl azides group in probe 2 was sensitive enough for thiols but azides’ instability character limited its application. The benzofurazan sulfide group of probe 3 appeared to react at a much slower rate with thiols and showed pH-dependent reactivity, but it is difficult to synthesis and modify due to its particularity structural. As shown in probe 4 and 5, 2,4-dinitrobenzenesulfonyl (DNBS) has excellent selectivity towards thiols with photoinduced electron transfer (PET) OFF–ON fluorescence switching effect and can link to fluorophores conveniently.

**Figure 1:**
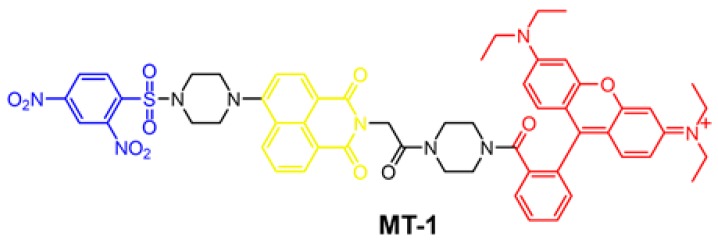
Chemical structure of MT-1.

In view of the aspects mentioned above, we think it is still necessary to develop a more sensitive, practicable and easily prepared fluorescent probe for mitochondrial thiols detection. Here we designed and synthesized a two-photon probe MT-1 based on two-photon and fluorescence resonance energy transfer (FRET) strategy [46,47], as shown in Figure 1. Naphthalimide was a widely used biocompatible fluorophore with large two-photon absorption cross sections [48,49], so we chose it for the two-photon receptor as well as the FRET donor. Then inspired by probe 4 and 5 mentioned above, we hired the rhodamine B for FRET acceptor and mitochondria-targeting group simultaneously. The FRET pairs were connected by a novel designed, simple and easily synthesized linker. Finally, DNBS was chosen as the response group and linked to the naphthalimide moiety by piperazine. The high-level electron deficiency of DNBS moiety enables its action as an electron sink and induced intramolecular charge transfer (ICT) effect, resulting in the quenching of the fluorescence [43,50]. Probe MT-1 was compact designed and easily synthesized with an extended emission wavelength (590 nm) and excitation wavelength (395 nm for one photon and 800 nm for two-photon) as well as great thiols sensitivity. By taking advantage of the superior properties of two-photon and FRET, we expected MT-1 can serve as an excellent mitochondrial thiols probe for live cells and tissue imaging. To the best of our knowledge, although fluorescent probes for biothiols have been widely developed, probes with the two-photon and Förster resonance energy transfer (TP-FRET) strategy for mitochondrial thiols have not been reported.

## 2. Materials and Methods

### 2.1. Materials and Instrumentation

All chemical reagents in the syntheses were purchased from Energy Chemical (Shanghai, China) Co., Ltd., and used without purification unless otherwise. Solvents were dried by standard methods prior to use. MitoTracker Green FM and 3-(4,5-Dimethylthiazol-2-yl)-2,5-diphenyltetrazolium bromide were all obtained from Thermo-Fisher Biochemical Products (Beijing, China) Co., Ltd. ^1^H NMR and ^13^C NMR spectra of synthetic intermediates and final probe were recorded on Agilent DD2 (Agilent Technologies, Santa Clara, CA, USA) (600 MHz, 150 MHz). Electrospray ionization mass spectrometry (ESI-MS) was operated on a Bruker maXis 4G mass spectrometer (Bruker Daltonik Gmbh, Bremen, Germany). pH measurements were made on a Sartorius PB-10 pH meter (Göttingen, Germany). UV-Vis absorption spectra were recorded on a SHIMADZU UV-1750 spectrophotometer (Kyoto, Japan). Fluorescence spectra were taken on a PerkinElmer LS-55 spectrofluorometric (Waltham, MA, USA). HeLa cells were cultured in a Thermo Forma, model 371 CO_2_ incubator (Thermo Fisher Scientific, Waltham, MA, USA). The absorbance for an MTT assay was obtained using a Thermo Scientific Varioska Flash microplate reader (Waltham, MA, USA). Confocal microscopy fluorescence images and two-photon fluorescence images were taken using an Olympus FV1000-MPE multiphoton laser scanning confocal microscope (Olympus Co., Ltd., Nagano, Japan).

### 2.2. Synthesis and Characterization

**Scheme 1:**
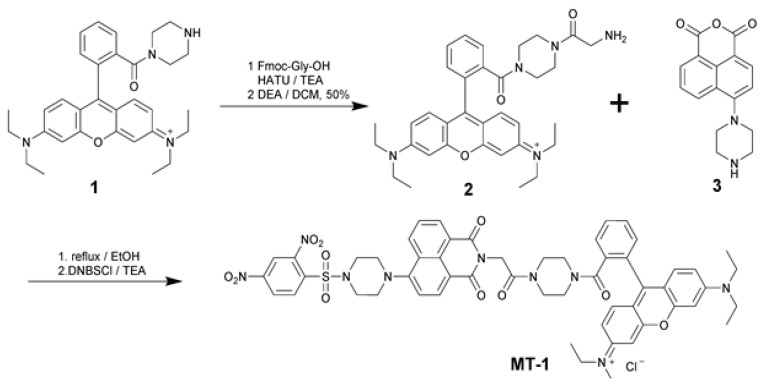
Synthetic route of MT-1.

The general synthetic route of MT-1 was shown in Scheme 1. Compounds **1** and **3** were synthesized according to the reported procedure [51,52]. First compound **1** (650 mg, 0.56 mmol), Fmoc-Gly-OH (756 mg, 2 eq.), HATU (966 mg, 2 eq.) and TEA (354 μL, 2 eq.) were dissolved in dry dichloromethane, then the mixture was stirred at room temperature overnight. After completion, 10 mL water was added, and the mixture was extracted with dichloromethane (DCM) (10 mL × 3), the organic phases were combined and dried over MgSO_4_, then concentrated to give a red solid without further purification. The solid was re-dissolved in 50% diethylamine/DCM (V/V) and stirred for 2 h. Then the solvent was acidified with 1M HCl, the aqueous phase washed with DCM for 3 times and then alkalified with 1M NaOH, after extracted with DCM (10 mL × 3), the organic phase was collected, washed with brine and dried over MgSO_4_, then concentrated to give Fmoc deprotected compound **2** as a red solid without further purification. ESI-MS: (C_34_H_42_N_5_O_3_^+^) calculated for 568.33, found m/z 568.33 [M]^+^. Compound **2** (51.3 mg, 0.09 mmol) and **3** (38.2 mg, 1.5 eq.) were dissolved in 5 mL EtOH, the mixture was refluxed overnight, then concentrated to give a red solid without further purification. Then the solid was re-dissolved in dry dichloromethane with TEA (12.5 μL, 1 eq.), stirred for 10 min, then a solution of 2,4-dinitrobenzenesulfonyl chloride (DNBSCl, 13.3 mg, 0.05 mmol) in dry 5 mL dichloromethane was added dropwise at 0 °C (ice-bath). The whole solution was stirred for another 10 h at room temperature. The solvent was removed under vacuum and the crude material was purified by flash chromatography on silica gel (dichloromethane/methanol, 10/1, V/V) to give MT-1 (49.8 mg, 33% yield for four steps). ESI-MS: (C_56_H_56_N_9_O_11_S^+^) calculated for 1062.38, found m/z 1062.37 [M]^+^. ^1^H NMR (600 MHz, CDCl_3_) δ (ppm): 0.86 (ddd, 3H), 1.20–1.23 (m, 6H), 1.32 (d, 7H), 3.36 (d, 8H), 3.59 (t, 4H), 3.70 (ddd, 8H), 4.93 (s, 2H), 6.71 (s, 1H), 6.83 (s, 1H), 6.88 (s, 1H), 7.17–7.21 (m, 1H), 7.33 (d, 1H), 7.37 (d, 1H), 7.63 (d, 5H), 8.21 (d, 2H), 8.33 (d, 1H), 8.57 (s, 3H). ^13^C NMR (151 MHz, CDCl_3_) δ (ppm): 157.70, 156.23, 155.72, 155.70, 155.32, 155.11, 149.31, 148.21, 148.20, 147.40, 145.17, 145.17, 132.76, 131.97, 131.87, 131.60, 131.51, 130.71, 130.29, 129.39, 129.38, 128.43, 127.94, 127.67, 127.05, 126.78, 125.25, 123.51, 119.92, 119.08, 119.06, 118.27, 113.22, 58.40, 58.39, 52.35, 50.80, 46.12, 29.66, 22.66, 18.40, 14.08, 12.64.

### 2.3. UV-vis and Fluorescence Measurements

Double distilled water was used to prepare all the aqueous solutions. Stock solution of MT-1 (1.0 mM) was prepared in dimethylsulfoxide (DMSO), and the solutions for spectroscopic determination were obtained by diluting the stock solution to a final concentration of 10 μM with 10% DMSO/ phosphate buffered saline (PBS) (V/V) (50 mM, pH = 7.4). Stock solutions (0.1 M) of the three biothiols and others amino acids were prepared in double distilled water. The spectra of the solutions were obtained by using a fluorescence spectrometer and the excitation wavelength was 395 nm. All spectroscopic experiments were carried out at 37 °C.

### 2.4. Determination of the Detection Limit

Calculation of detection limit was based on the fluorescence titration curve of probe MT-1 in the presence of biothiols. The fluorescence intensity of MT-1 was measured five times and the standard deviation of blank measurement was calculated. The detection limit was calculated with the following equation: Detection limit = 3σ/k, where σ is the standard deviation of the blank measurement (1.25 for GHS, 0.89 for Cys and 1.73 for Hcy), k is the slope between the fluorescence intensity versus GSH, Cys or Hcy concentrations (4.2 for GHS, 5.73 for Cys and 2,15 for Hcy).

### 2.5. Cell Culture and MTT Assay

The cellular cytotoxicity of MT-1 toward HeLa cells was evaluated using the standard methyl thiazolyl tetrazolium (MTT) assay. HeLa cells were seeded into a 96-well plate at a concentration of 4 × 10^3^ cells/well in 100 μL of dulbecco’s modified eagle medium (DMEM) medium with 10% fetal bovine serum (FBS). The plate was maintained at 37 °C in a 5% CO_2_, 95% air incubator for 24 h. After the original medium was removed, the HeLa cells were incubated with different concentrations of MT-1 (from the stock solution in Section 2.3 with different volumes, final concentrations: 1, 2 and 5 μM, containing DMSO as cosolvent, all final DMSO concentrations were adjusted to 1% with culture solvent, V/V) for 24 h. The cells only incubated with the culture solvent (containing 1% DMSO as cosolvent) served as the control. The cells were washed with PBS three times, and then 100 μL MTT solution (0.5 mg/mL in PBS) was added to each well and incubated for 4 h. Then the supernatant was removed gently. After the addition of DMSO (150 μL/well), the assay plate was shaken at room temperature for 10 min. The spectrophotometric absorbance of the samples was measured by using a Thermo Fisher-Varioska Flash microplate reader for optical density at 490 nm.

### 2.6. Fluorescence Imaging in Cells

For subcellular colocalization analysis of MT-1 staining, a commercially available mitochondrial localizing dye MitoTracker Green FM was used. HeLa cells were seeded on a 6-well plate at a density of 1 × 10^5^ in 2 mL of DMEM medium and then cultured at 37 °C under 5% CO_2_ for 24 h. After washing by PBS buffer, the cells were added to a DMEM medium containing 5 μM probe MT-1 (containing DMSO as cosolvent, final concentration is 1%, V/V) at 37 °C in an atmosphere of 5% CO_2_, 95% air incubator for 30 min, and then washed three times with PBS. Cells were incubated with MitoTracker Green FM (200 nM) at 37 °C under the same condition, and again washed with PBS three times. After the replacement of the medium, cells were imaged using an Olympus FV1000-MPE confocal fluorescence microscope.

For the two-photon fluorescent imaging experiments, HeLa cells were seeded on a 6-well plate at a density of 1 × 10^5^ in 2 mL of DMEM medium and then cultured at 37 °C under 5% CO_2_ for 24 h. After washing by PBS buffer, the cells were added to DMEM medium containing 5 μM probe MT-1 (containing DMSO as cosolvent, final concentration is 1%, V/V). After 30 min incubation, the cells were washed by PBS buffer again, and 100 μL PBS was given to each well. Fluorescent imaging was then performed using an Olympus FV1000-MPE confocal fluorescence microscope (800 nm). For the thiols-blocking experiment, the DMEM medium containing NEM (N-ethylmaleimide, a commonly used intracellular thiol scavenger) was added (1 mM) to cells, and the cells were cultured for 40 min, after being washed with PBS, and then incubated with 5 μM probe MT-1. For the fluorescence recovering experiment, the cells were pretreated with NEM (1 mM) for 40 min, then washed with PBS and incubated with 200 μM Cys for 30 min, finally 5 μM probe MT-1 was added and incubated for another 30 min. The other conditions remained the same as above.

### 2.7. Two-Photon Tissue Imaging Experiments

For two-photon tissue imaging, slices were prepared from the liver of a 2-week-old mouse. Slices were cut to 1 mm thickness by using a microtome in 50 mM PBS buffer (pH = 7.4). Slices were incubated with 5 μM probe MT-1 in PBS buffer with 95% O_2_ and 5% CO_2_ for 30 min at 37 °C. Slices were then washed with PBS buffer three times and transferred to glass-bottomed dishes. Mouse liver slices imaging was carried out using an Olympus FV1000-MPE confocal fluorescence microscope at a wavelength of 800 nm.

## 3. Results

### 3.1. Design and Synthesis of MT-1

**Figure 2:**
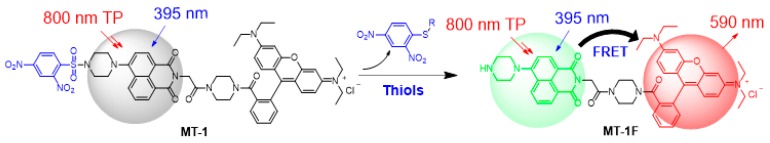
The proposed sensing mechanism of MT-1 with biothiols.

MT-1 is composed of three segments, first a widely used, biocompatible naphthalimide fluorophore as the FRET donor. Second, the positively charged rhodamine B fluorophore serves as a mitochondrial targeting site and FRET accepter simultaneously. Third, the widely used DNBS thiols detection group [22] afford an OFF–ON signal response for its unique sensitivity and high reactivity toward thiols. After reaction with thiols, the electron-withdrawing DNBS is removed and the fluorescence of MT-1 recovered through a FRET process (as shown in Figure 2). As have been thoroughly studied, the emission spectra of naphthalimide moieties exhibit a broad emission range which overlaps with the absorption spectra of rhodamine B and forms an ideal FRET pair [53,54,55]. We observed nearly 200 nm large pseudo-Stokes shift after the treatment of MT-1 with GSH (λ_ex_ = 395 nm, λ_em_ = 589 nm).

MT-1 was easily prepared through a four steps procedure without purification of intermediates. The general synthetic route was shown in Scheme 1 in which compounds **1** and **3** were synthesized according to the reported procedure [51,52]. Briefly, rhodamine B derivative **1** was condensed with Fmoc-Gly-OH followed by Fmoc deprotection to give **2,** which then was refluxed with **3** in ethanol and followed by reaction with DNBS-Cl in the presence of Et_3_N to give MT-1. The intermediate and final product was characterized by mass spectrometry, ^1^H NMR and ^13^C NMR (please see the supplementary materials).

### 3.2. Response of MT-1 to the Biothiols

**Figure 3:**
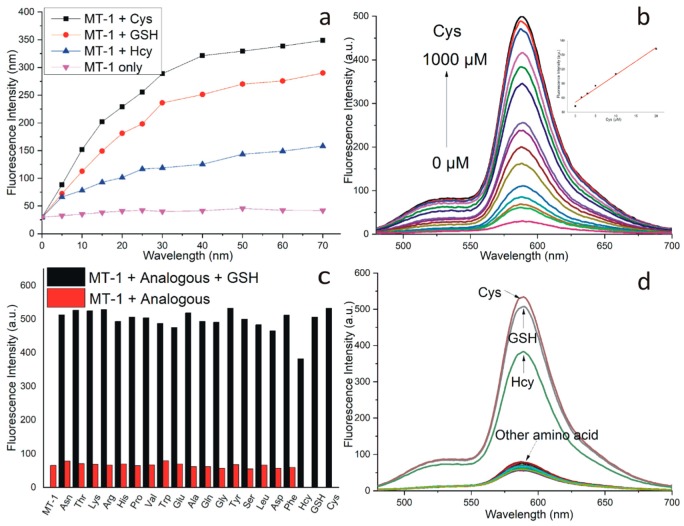
(**a**) Time-dependent fluorescence spectra of MT-1 (10 μM) upon addition of three biothiols (100 μM, respectively) in 10% DMSO/PBS (V/V) buffer (50 mM, pH = 7.4) at 37 °C. λ_ex_ = 395 nm, λ_em_ = 589 nm, slits (8, 8); (**b**) Fluorescence intensity of MT-1 (10 μM) incubated with different concentrations of Cys for 1 h under the same conditions as (a) and the linear relationship between the fluorescence intensity at 589 nm and concentrations of Cys (0–50 μM), slits (8, 8); (**c**), (**d**) Selectivity of the probe MT-1. Fluorescence intensity of MT-1 (10 μM) after incubation with biothiol (500 μM) for 1 h in the presence or absence of 17 amino acids (500 μM respectively) under the same conditions.

The proposed mechanism of MT-1 reaction with Cys was firstly investigated by mass spectrometry. As shown in Figure S3, after reaction with Cys, the mass 832.4378 was consistent with the generated FRET fluorophore MT-1F (calculated 832.42). The absorption spectra of MT-1 with thiols are shown in Figure S1, the absorption peaks at 570 nm correspond to the rhodamine B. After the addition of three thiols, the absorption peaks slightly change around 370 nm corresponded to the naphthalimide moieties. The time-dependent fluorescence spectral properties of MT-1 (10 μM) was first investigated. As shown in Figure 3a, upon addition of Cys in 10% DMSO/PBS (V/V) buffer (50 mM, pH = 7.4) at 37 °C, MT-1 displayed good kinetics for rapid detection of Cys and the fluorescence intensity increase to a plateau value within 30 min. Compared to Cys, the reaction of MT-1 with GSH and Hcy appeared to be slightly slower under the same condition.

Concentration-dependent study of MT-1 to three thiols was also investigated. As expected, the addition of Cys resulted in an elevated fluorescence intensity which became stronger with the increased Cys concentrations (Figure 3b). A good linear relationship between the fluorescence intensity at 589 nm and concentrations of Cys (ranging from 0 to 50 Μm, Y = 5.73X + 50.77, R = 0.984) was observed, and the detection limit was estimated to be 0.47 μM. Under these same conditions, MT-1 exhibited different fluorescence responses to GSH and Hcy with detection limits of 0.89 μM and 2.41 μM, respectively (see Figure S2).

We next investigated the selectivity of MT-1 for biothiols versus the other 17 amino acids. As illustrated in Figure 3c,d, in the presence or absence of potentially interfering species, MT-1 only reacted with three biothiols and showed higher fluorescence intensity, indicating that the probe exhibited higher selectivity for biothiols than other amino acids due to the unique sensitivity and high reactivity of DNBS toward thiolate.

**Figure 4:**
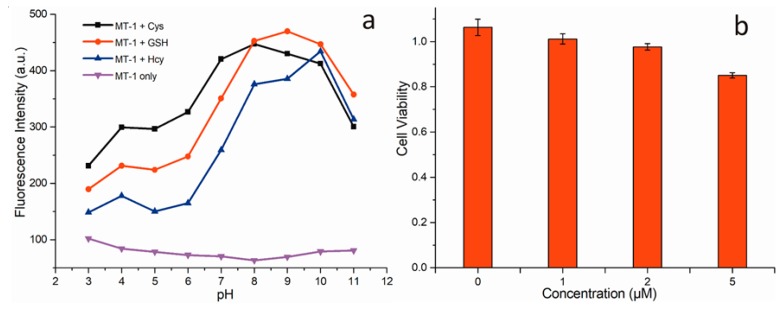
(**a**) Fluorescence intensity changes of the probe MT-1 (10 μM) at different pH values in the absence or presence of biothiols (100 μM, respectively) in 10% DMSO/PBS (V/V) buffer (50 mM, pH = 7.4) at 37 °C, λ_ex_ = 395 nm, λ_em_ =589 nm, slits (10, 10); (**b**) MTT assay with different concentrations of MT-1 (0-5 μM) at 37 °C for 24 h.

The effect of pH on the fluorescent behavior of MT-1 was also explored and it can be seen in Figure 4a, the fluorescence intensity of MT-1 is nearly unchanged over a wide range of pH from 3 to 11, indicating good stability of MT-1 in a wide pH range. However, after the addition of three biothiols to the solution of MT-1 at different pH values, the fluorescence intensity of MT-1 was significantly enhanced and a rapid increase from pH 6 to 8 was observed. This phenomenon was consistent with the first literature reporting that DNBS was a thiol reaction group [56]. The increased reaction rate of MT-1 at pH 6–8 was an advantage for imaging mitochondrial biothiols selectivity because the slow reaction at pH 6–8 could reduce the possibility of MT-1 to react with biothiols on its path to mitochondria, when it enters mitochondria, the alkaline environment would facilitate the reaction between MT-1 and biothiols [42].

### 3.3. Two-Photon Imaging of Thiols in Cell Lines

**Figure 5:**
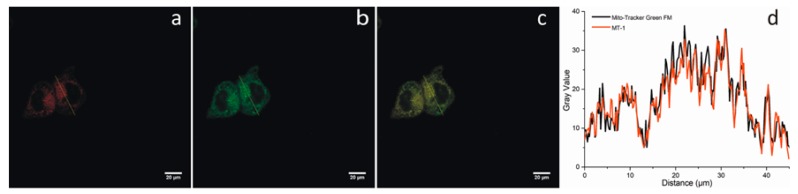
Colocalization of MT-1 and MitoTracker Green FM to mitochondria. HeLa cells were incubated with (**a**) 5 μM MT-1 for 30 min at 37 °C, red channel, λ_ex_ = 405 nm; (**b**) 200 nM MitoTracker Green FM for 30 min at 37 °C, green channel, λ_ex_ = 488 nm; (**c**) Overlay of (a) and (b); (**d**) Intensity profile within the regions of interest (short lines) across HeLa cells. scale bar: 20 μm.

Before the applications of MT-1 in HeLa cells for biothiols detection, standardMTT assay [57] was carried out to evaluate the cytotoxicity of MT-1. After incubation with 5 μM MT-1 for 24 h, the cellular viability of HeLa cells was estimated to be > 82%, indicating that MT-1 has low cytotoxicity (Figure 4b). To confirm whether MT-1 can specifically stain the mitochondria, the subcellular colocalization experiment of MT-1 was carried out, a commercially available mitochondria localizing dye MitoTracker Green FM was used for the co-localization study. As shown in Figure 5, the confocal fluorescence microscopy results showed that MT-1 (5 μM, 30 min) overlaid well with MitoTracker Green FM (200 nM, 30 min) with a Pearson correlation coefficient of r = 0.87 (calculated using ImageJ (NIH Image, https://imagej.net/), version: 1.52o), indicating that MT-1 exists predominantly in mitochondria.

**Figure 6:**
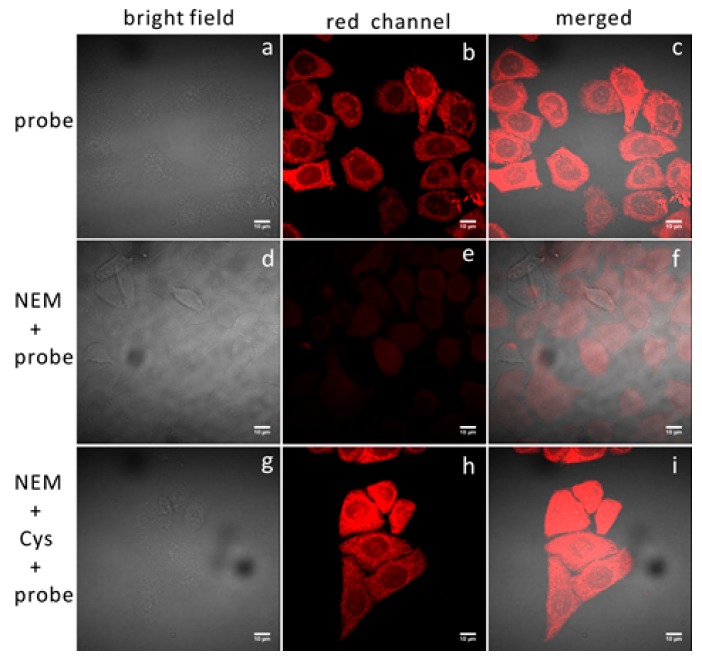
Two-Photon images of HeLa cells. (**a**, **b**, **c**) Cells incubated with only MT-1 (5 μM) for 30 min; (**d**, **e**, **f**) Cells incubated with NEM (N-ethylmaleimide) (1 mM) for 40 min before with MT-1 (5 μM) for 30 min; (**g**, **h**, **i**) Cells incubated first with NEM (1 mM) for 40 min, then Cys (200 μM) for 30 min and finally MT-1 (5 μM) for another 30 min. 37 °C, red channel, λ_ex_ = 800 nm, scale bar: 10 μm.

We next evaluated the capability of the probe MT-1 for mitochondrial biothiols detection in HeLa cells upon excitation at 800 nm (two-photon). As exhibited shown in Figure 6, when Hela cells were incubated with only MT-1 (5 μM) for 30 min, a strong fluorescence enhancement was observed. In contrast, when Hela cells were pre-treated with 1 mM NEM (N-ethylmaleimide, a commonly used intracellular thiol scavenger) before treated with MT-1 (5 μM), the fluorescence intensity was nearly vanishing, but a strong fluorescence was recovered when 200 μM Cys was added after treated with NEM, confirming unambiguously that the red fluorescence was induced by biothiols. This result shows that MT-1 can sense mitochondrial biothiols in living cells under two-photon excitation.

### 3.4. Two-Photon Tissue Imaging

**Figure 7:**
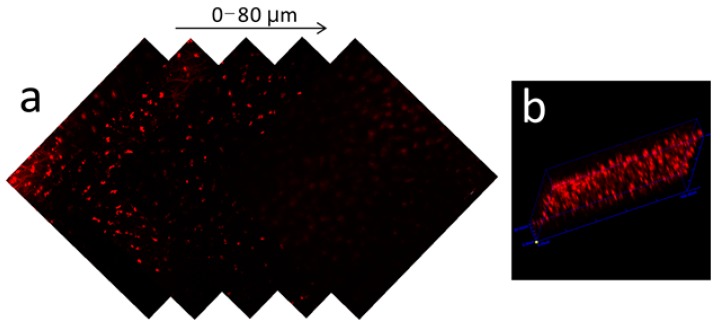
Two-Photon images of mouse liver slices stained with 5 μM MT-1 for 30 min at 37 °C. (**a**) The confocal z-scan TP imaging sections at different depths for 0 μm, 20 μm, 40 μm, 60 μm, 80 μm; (**b**) Three-dimensional reconstruction of the liver tissue images obtained before. Red channel, λ_ex_ = 800 nm. Scale bar: 50 mm.

Based on the result obtained above, we further performed fluorescence images of biothiols on mice liver slices to demonstrate the advantages of two-photon fluorescent probe MT-1. Freshly prepared liver tissue slices (thickness, 1 mm) of mice were treated with MT-1 for 30 min, followed by washing three times with PBS before imaged on TPM. An obvious red fluorescence signal was detected and collected at different tissue depths in the Z-scan mode and reconstructed in a three-dimensional (3D) box, as shown in Figure 7, the imaging depth of MT-1 was measured to be 80 μm. The results showed that MT-1 had good penetrability and staining ability for mitochondrial biothiols detection by the two-photon excitation mode.

## 4. Conclusions

In this paper, a new TP-FRET fluorescent probe MT-1 was constructed based on naphthalimide-rhodamine B FRET pairs for biothiols detection in the mitochondria of living cells. MT-1 was compact designed and easily synthesized. Due to the high reactivity of DNBS toward thiolate, MT-1 exhibited a fast response, high selectivity, and sensitivity to biothiols, as well as a low limit of detection. Compared to the reported probes, both excitation and emission wavelengths of MT-1 were extended. More important, the′s pH-dependent reactivity of MT-1 was a great advantage for selective detection of mitochondria biothiols. Two-photon cell imaging and tissue experiments revealed that MT-1 can serve as an effective tool to unravel the diverse biological functions of mitochondrial biothiols in living systems.

## Supplementary Materials

The following are available online at https://www.mdpi.com/1424-8220/20/6/1746/s1. Table 1: Reported probes for mitochondria thiols detection; Figure S1: Absorption spectra of MT-1; Figure S2: Fluorescence intensity and the linear relationship of MT-1 with different concentrations of GSH and Hcy; Figure S3: ESI-MS spectrometry of MT-1 upon addition of Cys; Figure S4: ESI-MS of **2**; Figure S5: ESI-MS of MT-1; Figure S6: 1H NMR of MT-1; Figure S7: 13C NMR of MT-1.
