# Supplementary material for: A FRET Based Two-Photon Fluorescent Probe for Visualizing Mitochondrial Thiols of Living Cells and Tissues
Source: Sensors (Basel). 2020 Mar 21;20(6):1746. doi: 10.3390/s20061746 (PMC7147317; doi:10.3390/s20061746)
Supplement: Supplementary file 1 [file sensors-20-01746-s001.pdf]

sensors

Supporting Information

A FRET Based Two-Photon Fluorescent Probe for Visualizing Mitochondrial Thiols of Living Cells and Tissues

Zhengkun Liu [1], Qianqian Wang [1], Hao Wang [1], Wenting Su [1] and Shouliang Dong [1,2,*]

1 Institute of Biochemistry and Molecular Biology, School of Life Sciences, Lanzhou University, 222 Tianshui South Road, Lanzhou 730000, China; liuzhk14@lzu.edu.cn (Z.L.); wangqq16@lzu.edu.cn (Q.W.); wangh2017@lzu.edu.cn (H.W.); suwt18@lzu.edu.cn (W.S.)

2 Key Laboratory of Preclinical Study for New Drugs of Gansu Province, Lanzhou University, 222 Tianshui South Road, Lanzhou 730000, China

* Correspondence: dongsl@lzu.edu.cn; Tel: +869318912428

Table of contents

1. Reported probes for mitochondria thiols detection Table 1
2. Absorption spectra of MT-1 Figure S1
3. Fluorescence intensity and the linear relationship of MT-1 with different concentrations of GSH and Hcy Figure S2
4. ESI-MS spectrometry of MT-1 upon addition of Cys Figure S3
5. ESI-MS of **2** Figure S4
6. ESI-MS of MT-1 Figure S5
7. ^{1}H NMR of MT-1 Figure S6
8. ^{13}C NMR of MT-1 Figure S7

Table 1. Probes for mitochondria thiols detection.

Numbers	Probes for mitochondria thiols detection	Journals	Strategies	Mechanism for selectivity mitochondrial thiol detection
1	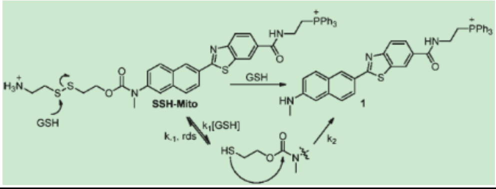	J. Am. Chem. Soc 2011.[1]	Two photon λ_{ex} = 740 nm λ_{em} = 545 nm	not mentioned
2	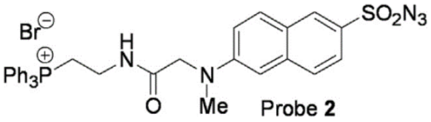	Dyes and Pigments 2013.[2]	Two photon λ_{ex} = 750 nm λ_{em} = 442 nm	not mentioned
3	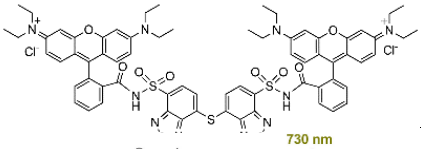	Anal. Chem 2018[3]	λ_{ex} = 550 nm λ_{em} = 580 nm	different pH between mitochondria and cytoplasm
4	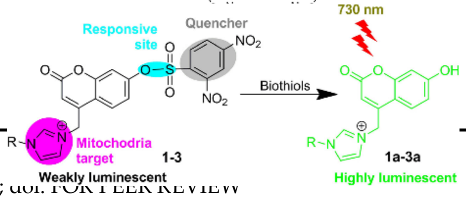	Sensors and Actuators B	Two photon λ_{ex} = 730 nm	not mentioned

		2017.[4]	λ_{em} = 482 nm	
5	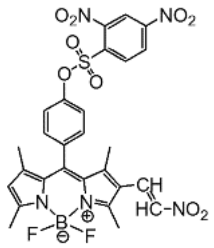	Dyes and Pigments 152 (2018) 29–35.[5]	λ_{ex} = 504 nm λ_{em} = 543 nm	not mentioned
This work	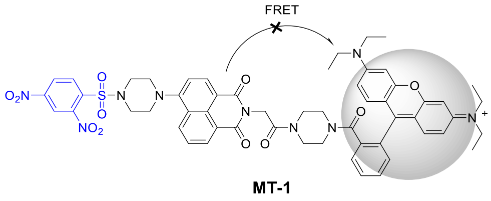	Our work	Two photon FRET λ_{ex} = 800 nm λ_{em} = 590 nm	pH dependent reactivity of probe

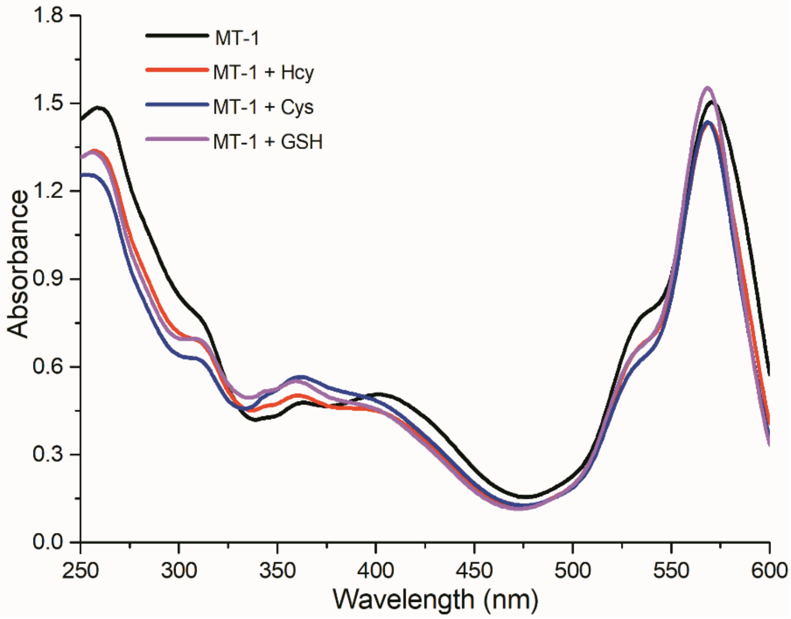

Figure S1. Absorption spectra of MT-1 before and after treatments with three biothiols (100 μM, respectively) in 10% (V/V) DMSO/PBS buffer (50 mM, pH = 7.4) at 37 °C.

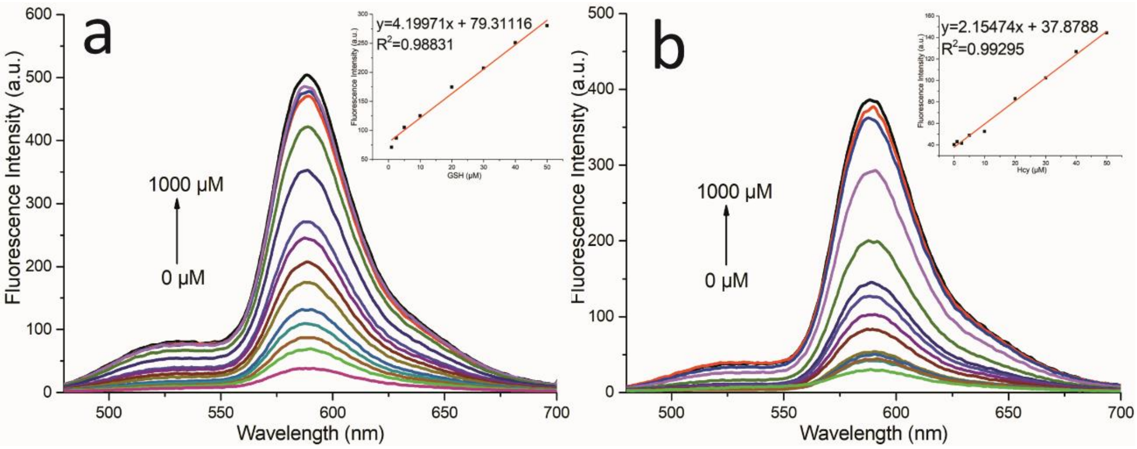

Figure S2. Fluorescence intensity and the linear relationship of MT-1 (10 μM) with different concentrations of GSH (**a**) and Hcy (**b**) for 1 hour in 10% (V/V) DMSO/PBS buffer (50 mM, pH = 7.4) at 37 °C. λ_{ex} = 395 nm, λ_{em} = 589 nm, slits (10, 10).

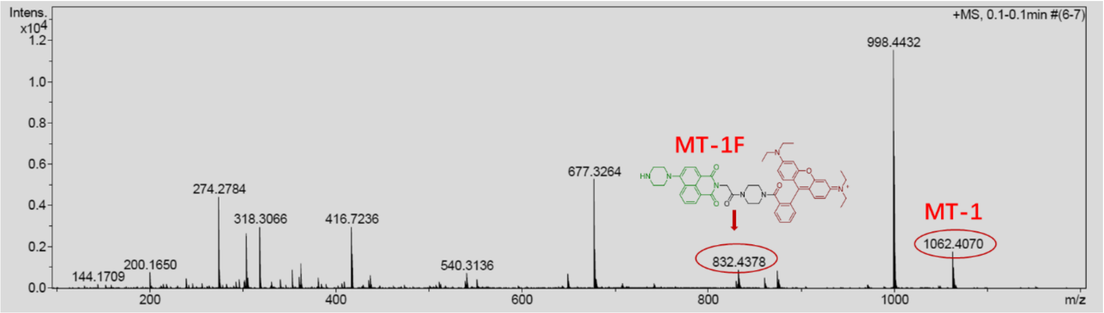

Figure S3. ESI-MS spectrometry of MT-1 upon addition of Cys in 10% DMSO/PBS (V/V) buffer (50 mM, pH = 7.4) at 37 °C.

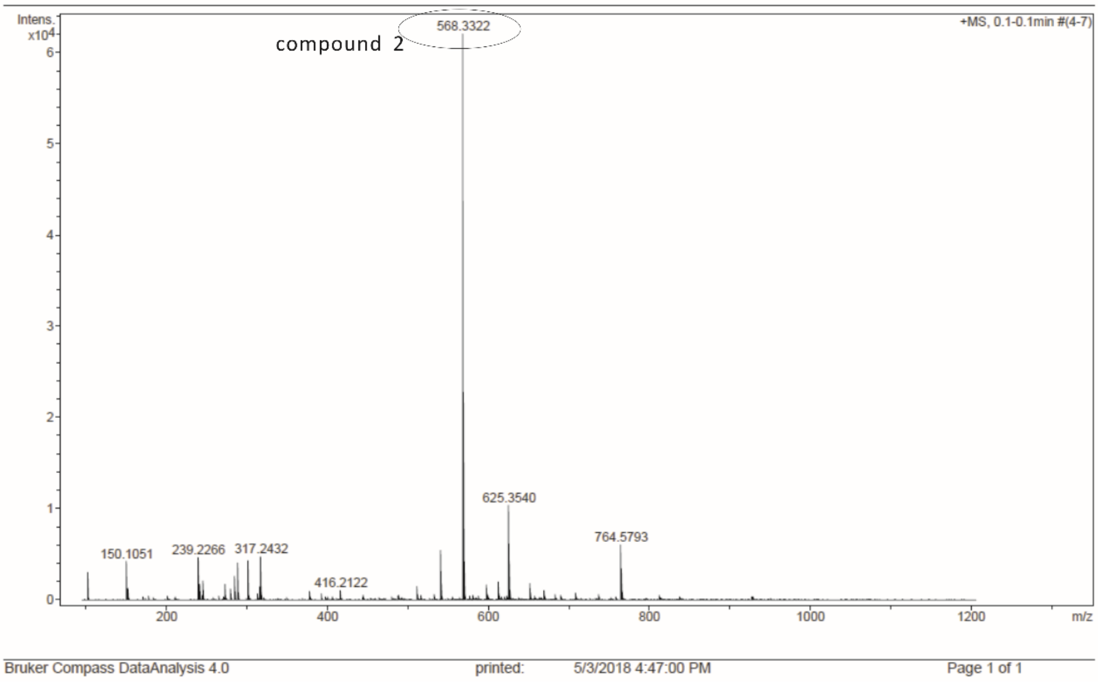

Figure S4. ESI-MS of **2.**

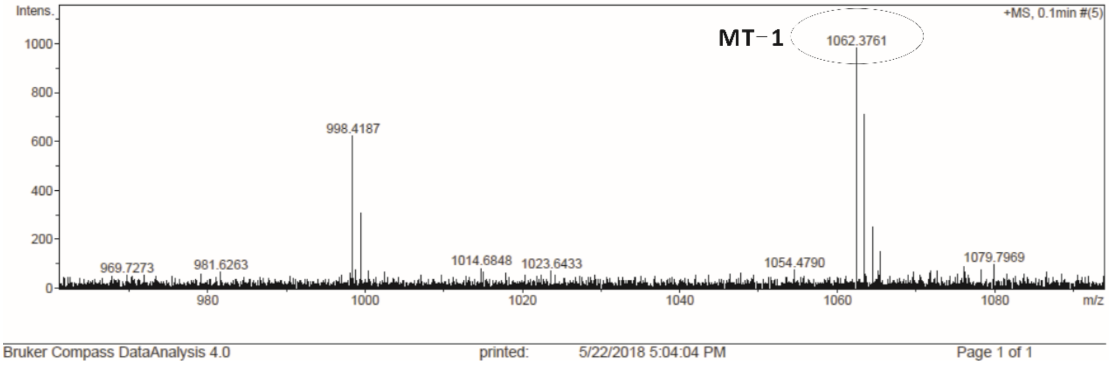

Figure S5. ESI-MS of MT-1.

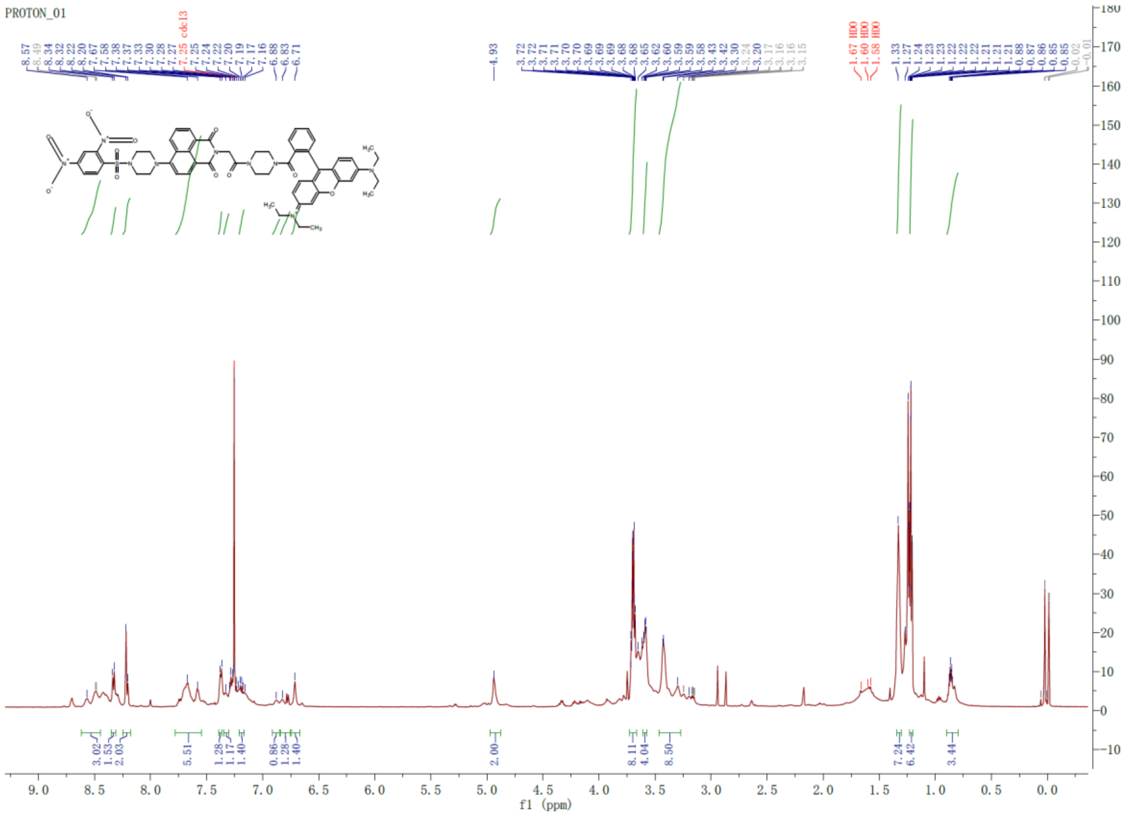

Figure S6. ^{1}H NMR of MT-1.

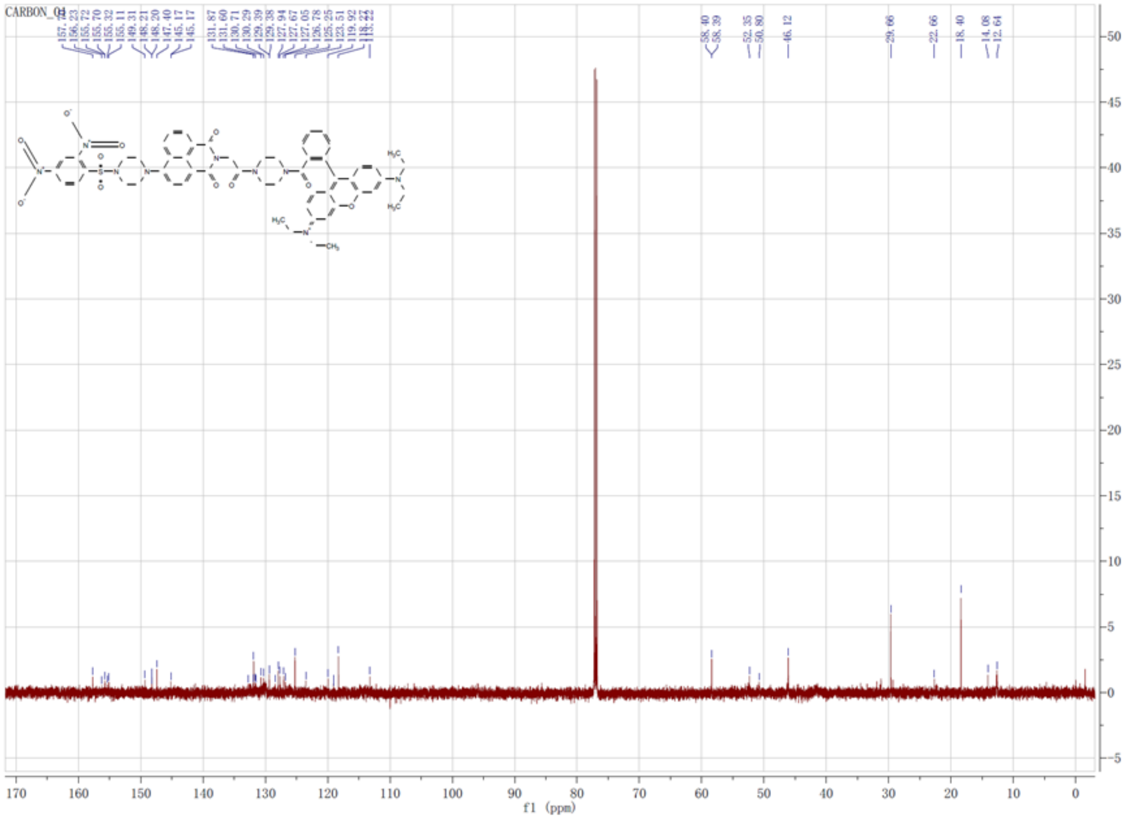

Figure S7. ^{13}C NMR of MT-1.

References

1. Su, L.C., et al., *Ratiometric detection of mitochondrial thiols with a two-photon fluorescent probe.* Journal of the American Chemical Society, 2011. **133**(29): p. 11132-5.
2. Singha, S., et al., *Two-photon probes based on arylsulfonyl azides: Fluorescence detection and imaging of biothiols.* Dyes & Pigments, 2013. **99**(2): p. 308-315.
3. Wang, S., et al., *Thiol Specific and Mitochondria Selective Fluorogenic Benzofurazan Sulfide for Live Cell Nonprotein Thiol Imaging and Quantification in Mitochondria.* Analytical Chemistry, 2018: p. acs.analchem.8b01469-.
4. Li, Y., et al., *Mitochondria-targeted two-photon fluorescent probe for the detection of biothiols in living cells.* Sensors & Actuators B Chemical, 2017. **255**: p. S0925400517314636.
5. Wang, F.-F., et al., *A BODIPY-based mitochondria-targeted turn-on fluorescent probe with dual response units for the rapid detection of intracellular biothiols.* Dyes and Pigments, 2018. **152**: p. 29-35.
